# The role of causal reasoning in understanding Simpson's paradox, Lord's paradox, and the suppression effect: covariate selection in the analysis of observational studies

**DOI:** 10.1186/1742-7622-5-5

**Published:** 2008-02-26

**Authors:** Onyebuchi A Arah

**Affiliations:** 1 Department of Social Medicine, Academic Medical Center, University of Amsterdam, PO Box 22700, 1100 DE Amsterdam, The Netherlands; 2 Department of Epidemiology, University of California, Los Angeles (UCLA), School of Public Health, Los Angeles, CA 90095-1772, USA

## Abstract

Tu *et al *present an analysis of the equivalence of three paradoxes, namely, Simpson's, Lord's, and the suppression phenomena. They conclude that all three simply reiterate the occurrence of a change in the association of any two variables when a third variable is statistically controlled for. This is not surprising because reversal or change in magnitude is common in conditional analysis. At the heart of the phenomenon of change in magnitude, with or without reversal of effect estimate, is the question of which to use: the unadjusted (combined table) or adjusted (sub-table) estimate. Hence, Simpson's paradox and related phenomena are a problem of covariate selection and adjustment (when to adjust or not) in the causal analysis of non-experimental data. It cannot be overemphasized that although these paradoxes reveal the perils of using statistical criteria to guide causal analysis, they hold neither the explanations of the phenomenon they depict nor the pointers on how to avoid them. The explanations and solutions lie in causal reasoning which relies on background knowledge, not statistical criteria.

## Commentary

Simpson's paradox, Lord's paradox, and the suppression effect are examples of the perils of the statistical interpretation of a real but complex world. By rearing their heads intermittently in the literature they remind us about the inadequacy of statistical criteria for causal analysis. Those who believe in letting the data speak for themselves are in for a disappointment.

Tu *et al *present an analysis of the equivalence of three paradoxes, concluding that all three simply reiterate the unsurprising change in the association of any two variables when a third variable is statistically controlled for [1]. I call this unsurprising because reversal or change in magnitude is common in conditional analysis. To avoid either, we must avoid conditional analysis altogether. What is it about Simpson's and Lord's paradoxes or the suppression effect, beyond their pointing out the obvious, that attracts the intermittent and sometimes alarmist interests seen in the literature? Why are they paradoxes? A paradox is a seemingly absurd or self-contradictory statement or proposition that may in fact be true [2]. What is so self-contradictory about the Simpson's, Lord's, and suppression phenomena that may turn out to be true? After reading the paper by Tu *et al *one still gets the uneasy feeling that the paradoxes are anything but surprising, that the statistical phenomenon they purport to represent are in fact causal in nature, requiring a causal language not a statistical one, and that the problem can be resolved only with causal reasoning. So, why bother with the statistics of these paradoxes, much less their equivalence, in the first instance if both the correct language and resolution lie elsewhere? Although we are given a glimpse of the appropriate tools (such as the implied causal calculus of directed acyclic graphs [3-6]), we must look beyond the authors' paper for satisfactory answers.

**Figure 1:**
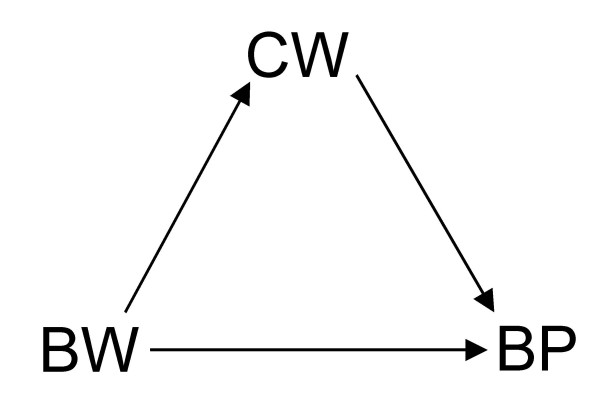
Directed acyclic graph (DAG) showing that birth weight (*BW*) has a direct effect as well as an indirect effect via current weight (*CW*) on blood pressure (*BP*).

**Figure 2:**
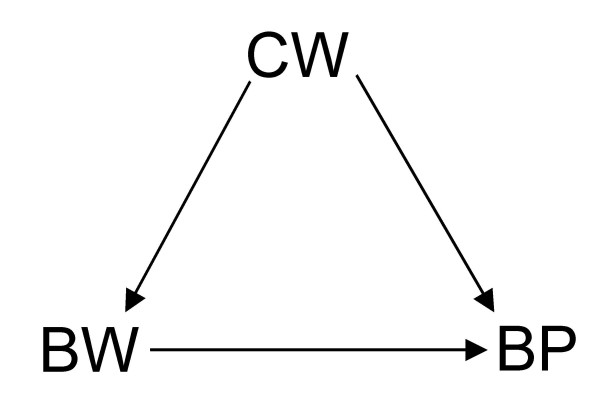
**DAG showing a scenario where birth weight (*BW*) has a causal effect on and shares a common cause – current weight (*CW*) – with blood pressure (*BP*).** That is, the relationship between *BW *and *BP *is confounded by *CW*.

**Figure 3:**
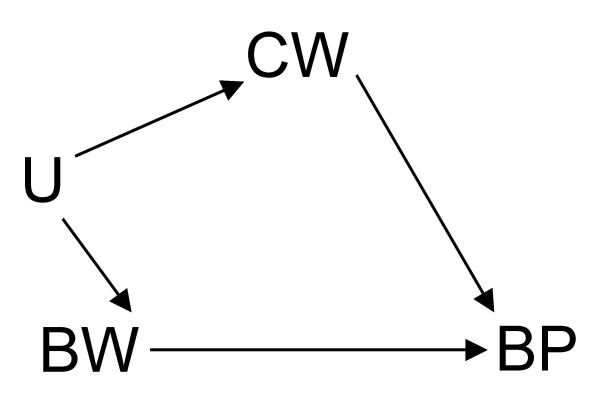
DAG showing where, in addition to its effect on blood pressure (*BP*), birth weight (*BW*) shares an unmeasured common cause (*U*) with current weight (*CW*) which also has an effect on *BP*.

**Figure 4:**
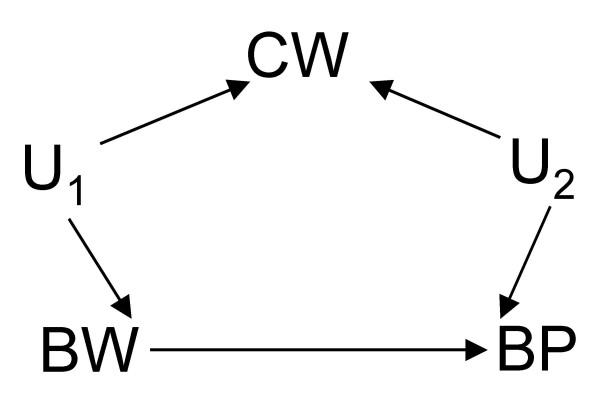
DAG showing where birth weight (*BW*) has a direct effect on blood pressure (*BP*) and shares an unmeasured common cause (*U*_1_) with current weight (*CW*) which itself has another unmeasured common cause (*U*_2_) with *BP*.

**Figure 5:**
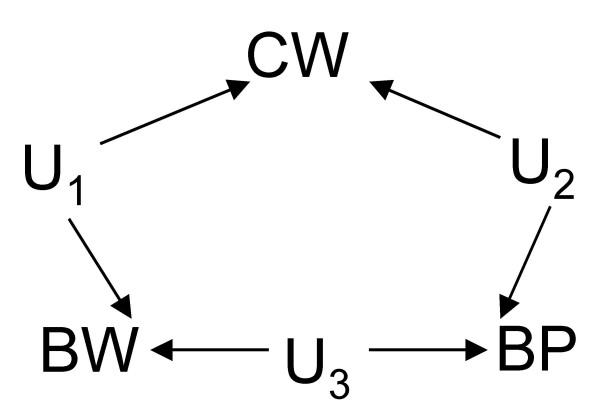
DAG depicting each variable pair – *BW*/*CW*, *CW*/*BP*, or *BW*/*BP *– as being connected only by an unmeasured common cause (*U*_1_, *U*_2 _or *U*_3 _respectively).

**Figure 6:**
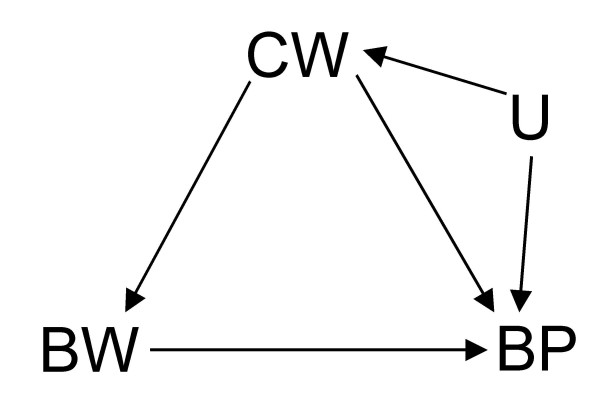
DAG where, like in Figure 2, the effect of birth weight (*BW*) on blood pressure (*BP*) is confounded by current weight (*CW*) which itself has an unmeasured common cause (*U*) with *BP*.

**Figure 7:**
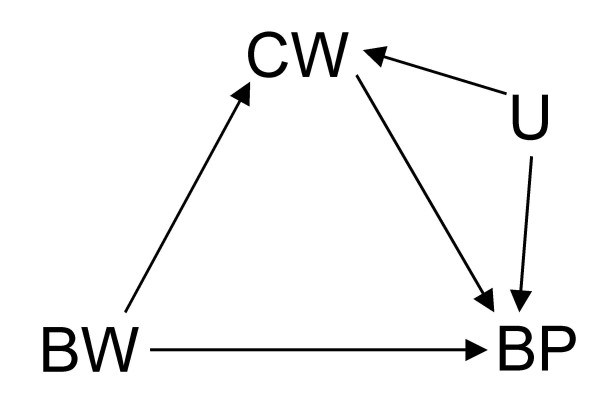
**DAG depicting a modification of Figure 1 where *CW *has a common cause (*U*) with *BP*.** In the directed path *BW*→*CW*←*U*→*BP*, *CW *acts as a collider if left uncontrolled for in the analysis of the effect of *BW *on *BP*.

At the heart of the phenomenon of change in magnitude, with or without reversal of effect estimate, is the question of which to use: the unadjusted (combined table) or adjusted (sub-table) estimate. Simpson's and Lord's paradoxes generate shock when change in direction or magnitude (or both) of an estimate is observed while we are thinking causes; we then start wondering which estimate is the correct one. Suppression effect in addition frets about model fit to assess the correctness of unadjusted versus adjusted estimates. In each case, the researcher is looking at statistics to tell her what she may not admit to: which estimate must she accord causal interpretation in what causal world? In other words, these paradoxes arise in the context of covariate selection, especially when looking to select variables for adequate control of confounding in causal analysis [5]. Causal diagrams and their related causal calculus have emerged as a mathematically rigorous approach to depicting causal relations among variables, making underlying causal assumptions transparent, and guiding the selection of a sufficient set of covariates for consistent effect estimation [3-6]. In all causal diagrams or directed acyclic graphs (DAGs), it is important to note that the missing arrows are very important: they connote what the researcher believes to be the absence of causal effects encoded by those missing arrows. The knowledge needed to draw these DAGs and to guide subsequent analysis resides outside the study data. Hence, there can be no causal inference without background knowledge [5,7]. To appreciate the causal approach to the paradoxes, consider the three-variable model of birth weight (BW), current weight (CW), and blood pressure (BP) used by the authors and seen in the life course epidemiology literature. In addition to the directed graphs presented by the authors, I have added a few additional, non-trivial, and non-redundant (although by no means exhaustive) graphs that could have generated their observed correlations (Figures 1 to 7).

Now, suppose there are no other unmeasured covariates given the DAGs in Figures 1 to 7. If Figure 1 is the true state of affairs, to estimate the *total *effect of BW on BP, the unadjusted analysis will suffice. If, however, Figure 2 or 3 applies, then the adjusted (that is conditional on CW) is needed to estimate the total effect of BW on BP. This is because conditioning on CW will block the back-door BW to BP: BW←CW→BP in Figure 2 or BW←U→CW→BP in Figure 3. The reader could by now have doubts about the correctness of Figure 2 where a later observation CW is a confounder of the effect of BW on BP since one could argue that, by occurring after BW, CW could not be seen as a common cause of both BW and BP. (See Hernán *et al *[8] for an accessible defence of the structural approach to confounding and selection bias using DAGs.) Nonetheless, while temporality *seemingly *excludes CW as a confounder in Figure 2, it does not exclude CW from ever being part of a confounding path as seen in Figure 3. Both BW and CW are more likely to be the result of a common cause (U), possibly genetic. Based on background knowledge and common sense, Figure 3 is more plausible than 2. Therefore, temporality cannot be used to judge whether a variable is a confounder, part of a sufficient subset of covariates needed to block a backdoor, or not [5].

Figure 4 presents a scenario where the unadjusted effect of BW on BP is the correct estimate since CW is a collider (that is, without conditioning, it already acts as a blocker) in the DAG depicting two unobserved common causes of BW and CW and of CW and BP. This scenario is closely related to that in Figure 5 where BW has no effect on, but shares an unobserved common cause (U_3_) with, BP. In all scenarios, our choice of which (unadjusted or adjusted) estimate to use is not based on the magnitude or direction of the estimate but on the governing causal relations. Put this way, Simpson's paradox becomes a problem of covariate adjustment (when to adjust or not) in the causal analysis of non-experimental or observational data. The paradox arises due to the causal interpretation of the observation that the proportion of a given level of BW is evidence for making an educated guess of the proportion of a given BP level in an observed sample *if *the status of the third related covariate CW is unknown [5]. What we really want to answer is "Does BW cause BP?", *not *"Does observing BW allow us to predict BP?".

As Pearl has noted [5], people think "causes", not proportions (the thing that drives the paradox in Simpson's paradox); "reversal" is possible in the calculus of proportions but impossible in the calculus of causes. Put in Pearl's causal language, the invariance of causal interpretation that is wrongly used to interpret evidence of reversal in proportions in Simpson's paradox is as follows:

Pr(BP=high | *do*{BW=high}, high CW) < Pr(BP=high | *do*{BW=low}, high CW)

Pr(BP=high | *do*{BW=high}, low CW) < Pr(BP=high | *do*{BW=low}, low CW)

where, according to our causal intuition, the combined or unadjusted analysis should be:

Pr(BP=high | *do*{BW=high}) < Pr(BP=high | *do*{BW=low})

The inequalities in (1), (2) and (3) reflect the "sure thing principle" which when applied to Tu *et al*'s paper would then go as follows: an action *do*{BW} which decreases the probability of the event BP in each CW subpopulation must also decrease the probability of BP in the whole population, provided that the action *do*{BW} does not change the distribution of the CW subpopulations. See Pearl [5] for a formal proof, although the sure thing principle follows naturally from the semantics of actions as modifiers of mechanisms, as embodied by the *do*(·) operator. What is numerically observed in Simpson's paradox, however, is

Pr(BP=high | BW=high) > Pr(BP=high | BW=low)

which goes against our causal intuition or inclination to think "causes". If the DAG represented in Figure 3 – or, for the sake of argument, Figure 2 – applies, then we must consult the conditional analysis represented by inequalities 1 and 2, not the observed unconditional analysis in inequality 4. In this context, inequality 4 can only be seen as an evidence of BP that BW provides in the absence of information on CW, not as a statement of the causal effect of BW on BP which is what inequality 3 captures [5]. That is, Simpson's paradox arises because once CW is unknown to us, and we observe, for instance, that the proportion of {BW=high} is higher than that of {BW=low}, we have evidence for predicting (as in inequality 4) that the observable proportion of {BP=high} would also be higher than that of {BP=low} in the non-experimental data, but we cannot take this observation to imply that {BW=high} causes {BP=high} which goes against our causal knowledge that *doing*{BW=low} causes {BP=high} as depicted in inequality 3. Hence, prediction does not imply aetiology. The former tends to deal with usually transitory proportions whereas the latter deals with invariant causal relations.

A further illustration of the futility of the continued statistical discussion of the paradoxes is captured in the discussion of the suppression effect: how an unrelated covariate (CW) "increases the overall model fit ...assessed by *R*^2^..." [1]. Tu *et al *should not be surprised that suppression is little known in epidemiology because epidemiologists do not and should not use the squared multiple-correlation-coefficient *R*^2 ^as a measure of goodness-of-fit. As Tu *et al *algebraically admit, *R*^2 ^is only an indication of the proportion of the variance in BP or outcome that is attributable to the variation in the fitted mean of BP [9]. It is known that the expected value of *R*^2 ^can increase as more and even unrelated variables are added to the model thus making it a useless criterion for guiding covariate selection [10].

Furthermore, Tu *et al *make a passing mention of direct effect versus indirect effect (as might be the case in the consideration of adjustment in Figure 1). This is, of course, beyond the scope of their paper and, therefore, my commentary. I refer the curious reader to the important work on the complex issues involved in the estimation of direct effect [3,5,11-14]. Suffice it to say that, in common situations where total effect estimation is possible, direct effect may be unidentifiable. For instance, although all effects of BW on BP can still be consistently estimated even in a scenario where there is an additional unobserved common cause (U) of CW and BP as in Figure 6 (modified from Figure 2), the direct effect of BW on BP cannot be identified without measuring U in Figure 7 which is a similar modification of Figure 1. Like Pearl [5] and Holland and Rubin [15], I take these paradoxes to be related to causal concepts which are, thus, best understood in the context of causal analysis.

In conclusion, it cannot be overemphasized that although Simpson's and related paradoxes reveal the perils of using statistical criteria to guide causal analysis, they hold neither the explanations of the phenomenon they purport to depict nor the pointers on how to avoid them. The explanations and solutions lie in causal reasoning which relies on background knowledge, not statistical criteria. It is high time we stopped treating misinterpreted signs and symptoms ('paradoxes'), and got on with the business of handling the disease ('causality'). We should rightly turn our attention to the perennial problem of covariate selection for causal analysis using non-experimental data.

## Competing interests

OAA is an associate faculty editor of the journal *Emerging Themes in Epidemiology *(ETE).
